# The evolution and expansion of RWP-RK gene family improve the heat adaptability of elephant grass (*Pennisetum purpureum* Schum.)

**DOI:** 10.1186/s12864-023-09550-8

**Published:** 2023-08-31

**Authors:** Yarong Jin, Jinchan Luo, Yuchen Yang, Jiyuan Jia, Min Sun, Xiaoshan Wang, Imran Khan, Dejun Huang, Linkai Huang

**Affiliations:** 1 Herbivorous Livestock Research Institute, Chongqing Academy of Animal Sciences, Chongqing, 402460 China; 2 College of Grassland Science and Technology, Sichuan Agricultural University, Chengdu, 611130 China; 3 State Key Laboratory of Grassland Agro-Ecosystems, Key Laboratory of Grassland Livestock Industry Innovation, Ministry of Agriculture, College of Pastoral Agriculture Science and Technology, Lanzhou University, Lanzhou, 730020 China

**Keywords:** Adaptive evolution, *Pennisetum purpureum*, RWP-RK gene family, Whole genome doubling (WGD)

## Abstract

**Background:**

Along with global warming, resulting in crop production, exacerbating the global food crisis. Therefore, it is urgent to study the mechanism of plant heat resistance. However, crop resistance genes were lost due to long-term artificial domestication. By analyzing the potential heat tolerance genes and molecular mechanisms in other wild materials, more genetic resources can be provided for improving the heat tolerance of crops. Elephant grass (*Pennisetum purpureum* Schum.) has strong adaptability to heat stress and contains abundant heat-resistant gene resources.

**Results:**

Through sequence structure analysis, a total of 36 RWP-RK members were identified in elephant grass. Functional analysis revealed their close association with heat stress. Four randomly selected RKDs (RKD1.1, RKD4.3, RKD6.6, and RKD8.1) were analyzed for expression, and the results showed upregulation under high temperature conditions, suggesting their active role in response to heat stress. The members of RWP-RK gene family (36 genes) in elephant grass were 2.4 times higher than that of related tropical crops, rice (15 genes) and sorghum (15 genes). The 36 *RWPs* of elephant grass contain 15 *NLPs* and 21 *RKDs*, and 73% of *RWPs* are related to WGD. Among them, combined with the DAP-seq results, it was found that RWP-RK gene family expansion could improve the heat adaptability of elephant grass by enhancing nitrogen use efficiency and peroxidase gene expression.

**Conclusions:**

RWP-RK gene family expansion in elephant grass is closely related to thermal adaptation evolution and speciation. The RKD subgroup showed a higher responsiveness than the NLP subgroup when exposed to high temperature stress. The promoter region of the RKD subgroup contains a significant number of MeJA and ABA responsive elements, which may contribute to their positive response to heat stress. These results provided a scientific basis for analyzing the heat adaptation mechanism of elephant grass and improving the heat tolerance of other crops.

**Supplementary Information:**

The online version contains supplementary material available at 10.1186/s12864-023-09550-8.

## Background

Since 1980s, the global climate has imposed serious impacts on the environment, especially high temperatures have been affecting crop yield and reducing arable land worldwide [1]. High temperature is one of the major factors affecting plant reproductive development and is also a driving force for plant evolution. Research has shown that heat stress can impact plant chromosome pairing, leading to natural polyploidy [2]. African regions “tropical continent” contains rich varieties of genetic resources to study long-term adaptive evolution in plants under heat stress [3]. Elephant grass (*Pennisetum purpureum* Schum., AABB, 2n = 4x = 28), from Africa commonly known as Napier grass which is cultivated worldwide as energy and forage grass due to its high biological yield and strong heat stress tolerance ability [4, 5].

The RWP-RK gene family consists of two subfamilies: NIN-Like Protein (NLP) and RWP domain-containing (RKD) are specific to plants and function as transcription factors [6]. It has been discovered in recent years that *NLP* plays a crucial role in answering nitrogen stress and regulating plant nitrogen metabolism [7]. This gene family is widely found in *Arabidopsis thaliala* [8], *Oryza sativa* [9], *Brachypodium distachyum* [10], *Triticum aestivum* [6] and *Zea mays* L. [11]. In addition, *NLP* showed responses against different types of abiotic stresses in rice and *Arabidopsis thaliana*, among which significant increase in *AtNLP4* and *AtNLP9* expression pattern was found under heat stress [12].

It has been reported that *RKD* involves in gametophyte growth, while compared with *NLP* studies in plants, its functions are relatively less studied in plant abiotic stress research [13, 14]. *Arabidopsis thaliana* has five *RKD* genes, *AtRKD4* is preferentially expressed in early embryos (several stages of development, such as seeds), and the deletion of this gene leads to abnormal development of fertilized egg cells [15]. In wheat, the expression of *TaRKD1* and *TaRKD2* in egg cells was detected by single-cell RT-PCR [16]. Specific expression of *CitRWP*, a homolog of Arabidopsis thaliana *RKD*, was observed in the ovules of *Citrus sinensis*, with higher expression in polyembryonal varieties compared to monoembryos, indicating that the gene exerts a critical effect on the development of nucellus embryo and apomixis in citrus [17]. In order to reveal the relationship between heat adaptation evolution and the expansion of the RWP-RK gene family in elephant grass, we conducted a systematic analysis of the RWP-RK gene family. By analyzing the evolution of the RWP-RK gene family and its response mechanisms under abiotic stress, we have provided new insights for studying plant heat tolerance.

## Results

### *RWPs* in elephant grass

In the elephant grass genome, 36 genes were identified to contain the RWP conserved domain after removing redundant sequences. Among these genes, 15 belong to the NLPs and 21 belong to the RKDs. After performing a phylogenetic analysis to avoid any confusion and overlapping gene names, the genes were renamed as *CpNLPs* and *CpRKDs* and ranked based on the subfamily to which they belonged, as presented in Table 1. The amino acid content of CpNLP protein ranges from 719 (*CpNLP6.2*) to 1047(*CpNLP3.4*), with relative molecular weights of 75.79 and 114.4 kDa. The CpRKD protein contains amino acids ranging from 194 (*CpRKD6.4*) to 865 (*CpRKD6.6*) with relative molecular weights of 21.81 kDa and 96.7kDA, respectively. The *C*p*NLP* contains more amino acids and has a larger molecular weight because it has one more Phox and Bem1 (PB1) domains than *CpRKD*. Interestingly, 93% of the *C*p*NLP* protein theoretical isoelectric point (pI) was less than 7, while 51% of the *CpRKD* protein theoretical pI was greater than 7. 94% of the *RWPs* through BUSCA (http://busca.biocomp.unibo.it, [18]) for subcellular localization prediction are expressed in the cell nucleus, except for *CpRKD6.6* and *CpRKD1.1* expressed in the cell membrane and chloroplasts.

**Table 1:** Characteristics of the elephant grass RWPs genes

Sequence ID	Gene ID	Amino Acid	MW	pI	Grand Average of Hydropathicity	Subcellular location
CpA0704442.1	Cp*NLP*3.1	933	102.485	5.75	-0.345	nucleus
CpB0105499.1	Cp*NLP*3.2	740	81.2685	6.26	-0.241	nucleus
CpB0105522.1	Cp*NLP*3.3	798	88.1575	6.86	-0.275	nucleus
CpB0200044.1	Cp*NLP*3.4	1047	114.419	5.93	-0.424	nucleus
CpB0202036.1	Cp*NLP*5.1	881	96.757	5.96	-0.35	nucleus
CpB0302056.1	Cp*NLP*1.1	877	96.3174	6.2	-0.353	nucleus
CpB0500258.1	Cp*NLP*1.2	895	99.9095	5.51	-0.449	nucleus
CpB0600353.1	Cp*NLP*6.1	719	76.1363	5.38	-0.243	nucleus
CpB0700826.1	Cp*NLP*2.1	942	102.565	5.79	-0.442	nucleus
CpA0104988.1	Cp*NLP*3.5	862	95.1375	6.33	-0.251	nucleus
CpA0201584.1	Cp*NLP*4.1	845	92.7198	6.05	-0.448	nucleus
CpA0300911.1	Cp*NLP*5.2	947	105.489	7.32	-0.37	nucleus
CpA0400456.1	Cp*NLP*6.2	719	75.794	6.18	-0.342	nucleus
CpA0501766.1	Cp*NLP*2.2	875	95.5893	5.88	-0.39	nucleus
CpA0600207.1	Cp*NLP*1.3	753	83.309	5.94	-0.441	nucleus
CpA0704451.1	*CpRKD*1.1	358	37.8142	9.45	-0.591	chloroplast
CpB0105588.1	*CpRKD*1.2	391	41.4397	5.2	-0.454	nucleus
CpB0200033.1	*CpRKD*1.3	277	29.3888	7.01	-0.484	nucleus
CpB0203114.1	*CpRKD*3.1	393	43.399	5.93	-0.468	nucleus
CpB0500039.1	*CpRKD*1.4	323	34.4816	9.6	-0.711	nucleus
CpB0502479.1	*CpRKD*6.1	251	28.4793	5.27	-0.577	nucleus
CpB0600386.1	*CpRKD*8.1	643	69.502	6.41	-0.266	nucleus
CpB0602860.1	*CpRKD*6.2	135	15.4887	8.48	-0.764	nucleus
CpB0602861.1	*CpRKD*6.3	244	28.2232	6.13	-0.643	nucleus
CpB0701300.1	*CpRKD*4.1	331	37.9678	9.03	-0.577	nucleus
CpB0701333.1	*CpRKD*4.2	346	39.3851	8.76	-0.607	nucleus
Cp0001542.1	*CpRKD*1.5	327	35.5405	6.15	-0.456	nucleus
CpA0100192.1	*CpRKD*6.4	194	21.8181	7.59	-0.262	nucleus
CpA0101264.1	*CpRKD*6.5	282	32.9897	6.25	-0.742	nucleus
CpA0105120.1	*CpRKD*1.6	355	37.8268	5.58	-0.481	nucleus
CpA0105163.1	*CpRKD*1.7	334	36.2957	7.79	-0.401	nucleus
CpA0401955.1	*CpRKD*6.6	865	96.7022	6.38	-0.285	organelle membrane
CpA0504624.1	*CpRKD*6.7	241	27.7356	8.23	-0.71	nucleus
CpA0600014.1	*CpRKD*1.8	277	29.3118	8.94	-0.592	nucleus
CpA0603907.1	*CpRKD*4.3	246	27.4974	9.64	-0.711	nucleus
CpA0702770.1	*CpRKD*3.2	373	41.2497	5.24	-0.39	nucleus

*MW* molecular mass(kDa), *pI* theoretical isoelectric point

### Expansion of the *RWPs* in *Pennisetum*

**Fig. 1:**
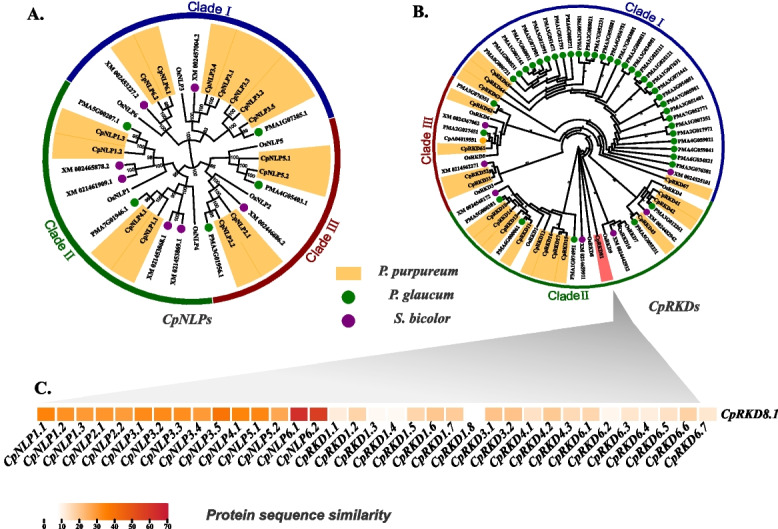
Phylogenetic analyses of RWPs in pearl millet, elephant grass, sorghum, and rice. **A** Phylogenetic analysis of NPLs proteins; **B** Phylogenetic analysis of RKDs proteins; **C** Sequence similarity comparison between CpRKD8.1 and CpNLPs

The full-length protein sequences of elephant grass (*Pennisetum purpureum* Schum.), sorghum (*Sorghum bicolor*), rice (*Oryza sativa*), and pearl millet (*Pennisetum. glaucum*) were aligned to reveal the phylogenetic relationship of *RWPs* in elephant grass. In addition, to more clearly compare the evolutionary differences between *RWPs* in elephant grass and other species, we carried out phylogenetic analyses on the *NLP* and *RKD* subfamilies of RWP-RK gene family (Fig. 1). The 31 NLPs were classified into three major clades consisting of 12, 8, and 12 proteins, which is consistent with the previous evolutionary analysis (Fig. 1A). Compared with sorghum, the *NLPs* of *Pennisetum* (pearl millet and elephant grass) were more widely distributed, especially forming specific clades in Clade I. In addition, *NLPs* of subgenome A in elephant grass had higher homology with pearl millet and were distributed evenly in all branches.

Different from the clustering results of *NLPs and RKDs* of elephant grass could not clearly distinguish gene categories (Fig. 1B). Here, we found that *CpRKD8.1* in elephant grass was significantly different from other *CpRKDs* and formed a separate branch. Comparing *CpRKD8.1* with all *RWPs* in elephant grass, it was found that *CPRKD8.1* was more similar to *NLPs* in elephant grass, especially *CpNLP1.1* and *CpNLP1.2* (Fig. 1C). This may be related to the evolution of *RWPs* in elephant grass.

### WGD promoted *RWPs* gene sequence recombination and membership increase

Due to the high similarity between the sequences of *CpRKD8.1* and *NLPs* in elephant grass, to further analyze the evolutionary relationship between *RKDs* and *NLPs* in elephant grass, phylogenetic analysis was conducted on the *RWP* conserved domain sequences of 36 *RWPs* genes in elephant grass (Fig. S1). In elephant grass, *RKDs* and *NLPs* had independent branches, and the clustering results of *RKDs* showed an evolutionary trend from *CpRKD1* to *CpRKD6*, while *NLPs* formed two independent evolutionary directions, *CpNLP3* and *CpNLP1*. This may indicate a functional divergence between *RKDs* and *NLPs* under environmental selection. By analyzing the distribution of RWP gene family on elephant grass chromosomes, it was found that *CpRKD8.1* and *CpNLP6.1* were located on chromosome 6B and closely related (Fig. S2B). Therefore, the production of *CpRKD8.1* may be related to the self-replication of *CpNLP6.1* caused by WGD.

Because the differentiation time of sorghum was earlier than elephant grass and pearl millet, we analyzed the WGD influence of *RWPs* gene evolution in elephant grass by collinearity analysis between elephant grass and sorghum. MCScanX analysis identified 26 *RWPs* associated with WGD, including 15 *RKDs* and 11 *NLPs* (Fig. S2A). Interestingly, the distribution of *RWPs* related to WGD in elephant grass B and A subgenomes was also 15 and 11 (Fig. S2B), See Table S1 for details. In addition, a total of 29 gene pairs were found by collinearity analysis between elephant grass and sorghum, and all gene pairs of Ka/ KS < 1, suggesting that *RWPs* in elephant grass are highly conserved and subject to strong purification selection.

### WGD promoted the increase of *RKDs* and *NLPs* sequence diversity

Although *RWPs* are strongly selected for purification in elephant grass, phylogenetic analysis of *NLPs* and *RKDs* showed that *CpNLPs* are more conserved than *CpRKDs*. Therefore, to further analyze the functional differences between *NLPs* and *RKDs* in elephant grass, we analyzed the sequence structure of *NLPs* and *RKDs* respectively.

**Fig. 2:**
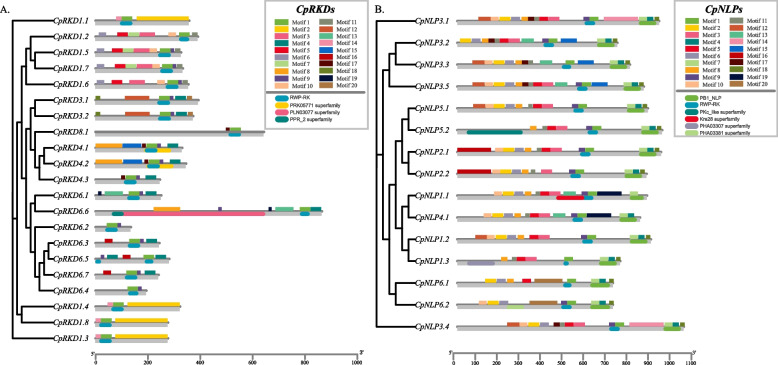
Motif analysis of RWPs in Elephant grass. **A** Motif analysis of RKDs protein sequences in Elephant grass; **B** Motif analysis of NLPs protein sequences in Elephant grass

The motifs of *NLPs* and *RKDs* genes in the elephant were visualized using the Simple MEME Wrapper tool in TBTools [19]. Twelve conserved motifs were identified from *CpNLPs*, including motif 9 and motif 1, which were identified as the N-terminal and C-terminal of *RWP* conserved motif by NCBI analysis (Fig. 2B). In addition, Motif7 and Motif4 were identified as the N-and C-terminus of the PB1 conserved area from 15 *CpNLPs*. Compared with *CpNLPs*, the amino acid sequences of *RKDs* showed greater diversity, with only 3 conserved motifs identified among 21 *RKDs* (Fig. 2A). Unlike *CpNLPs*, the *RWP* conserved domain (Motif1) of *RKDs* was well conserved in all genes, although *RKDs* showed large differences in amino acid sequences. In general, *NLPs* and *RKDs* in elephant grass changed due to the WGD sequence, which may be related to the selection of living environment and the adaptive evolution of genes.

### ABA and MeJA affect *RWPs* expression in elephant grasses under abiotic stress

**Fig. 3:**
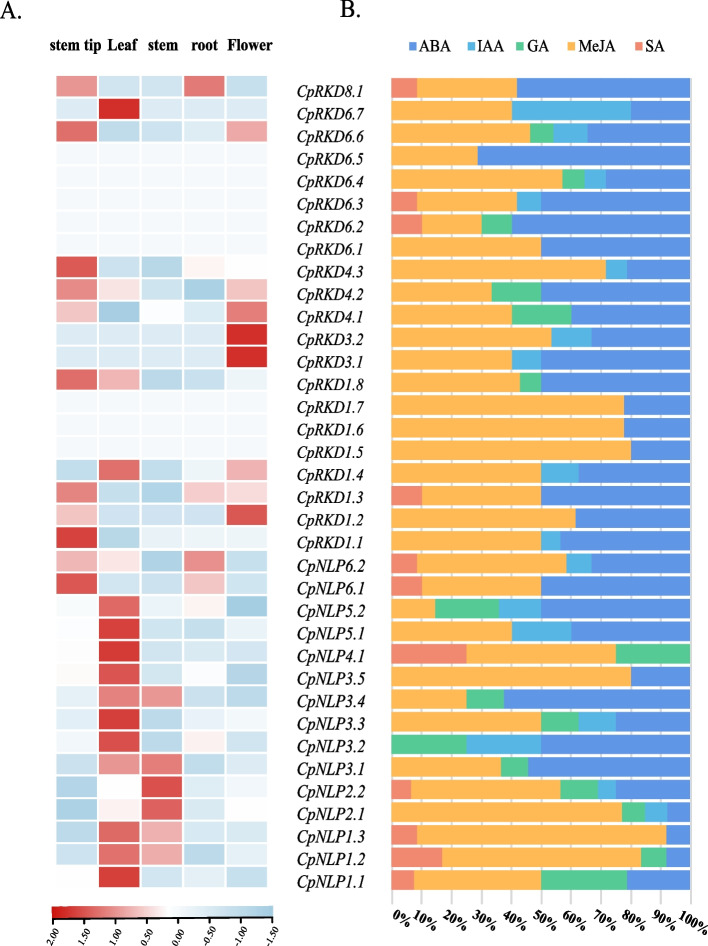
Statistical analysis of RWPs expression patterns (stem tip, leaf, stem, root, and flower) and promoter hormone response elements in elephant grass. **A** Expression of RKDs and NLPs in elephant grass 5 tissues **B** Analysis of CpRKDs and CpNLPs in response to hormone cis-acting elements

**Fig. 4:**
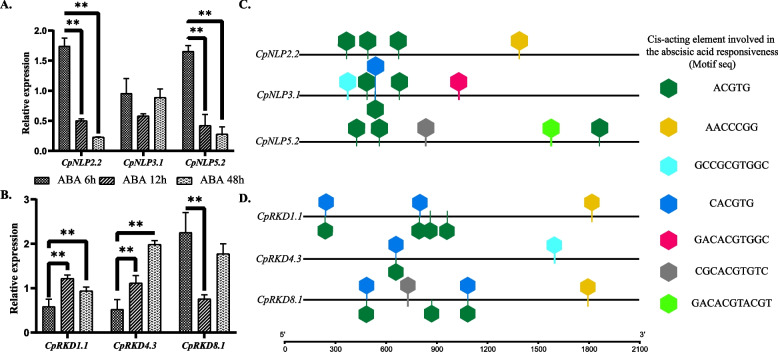
Effects of ABA on RWPs expression in elephant grass. **A** Expression trends of NLPs in Elephant grass at 6, 12 and 48 h after ABA (100 mM) treatment; **B** Expression trends of RKDs in Elephant grass at 6, 12 and 48 h after ABA (100 mM) treatment; **C** Distribution of abscisic acid-responsive elements in promoters (upstream < 2100) CpNLP2.2, CpNLP3.1 and CpNLP5.2 in elephant grass; **D** Distribution of abscisic acid elements in CpRKD1.1, CpRKD4.3 and CpRKD8.1 promoters (upstream < 2100) in elephant grass. (*: *p* < 0.05; ** *p* < 0.01)

*CpNLPs* were expressed (0.02–32.61 TPM) in all tissues (stem tip, leaf, stem, root, and flower), mainly in leaves and stem, and the highest expression levels in leaves and stems were *CpNLP3.1* and *CpNLP2.2*, respectively (Fig. 3B). Compared with *CpNLPs*, the expression of *CpRKDs* is more specific, and some genes are not detected in various tissues, which may be activated in response to specific environmental signals or signals at developmental stages (Fig. 3A). In addition, we found that *CpNLPs* and *CpRKDs* of elephant grass A subgenome had expression advantages, and the expression of *NLPs* and *RKDs* in elephant grass A subgenome was higher than that of genes belonging to B subgenome in all Clade (Table S2). According to plantcare cis-acting factors prediction, *RWPs* in elephant grass responded to a variety of phytohormone signals (IAA, GA, MeJA, BHA and ABA), MeJA and ABA had the most binding sites. In addition, by comparing the differences in hormone signal binding sites between *CpRKDs* and *CpNLPs*, it was found that *CpRKDs* contained more MeJA and ABA signal binding sites. Among all the cis-acting elements in response to hormones, the type of element that responded to ABA was the largest (Table S3). The expression trend of *RWPs* in elephant grass was detected by external application of ABA (100 mM) and qRT-PCR, and it was found that external application of ABA could affect its expression trend (Fig. 4).

**Fig. 5:**
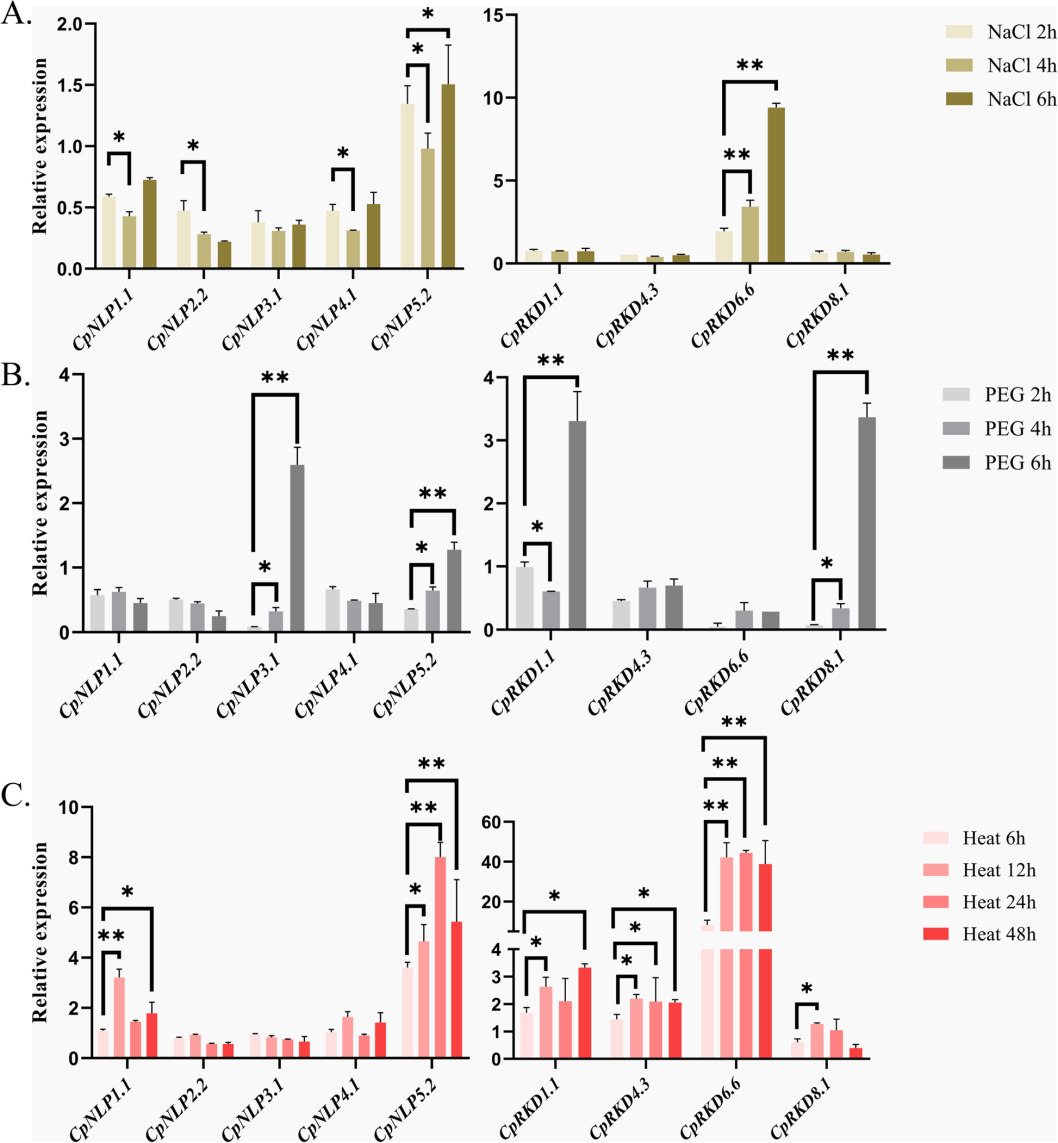
Expression trend of RWPs in elephant grass under abiotic stress (*:*p* < 0.05; **: *p* < 0.01). **A** salt (NaCl 100 mM; **B** drought(PEG 20%); **C** heat (40 °C/38 °C, 12 h light / 12 h dark). (*:*p* < 0.05; ** *p* < 0.01)

For further analyzing the expression differences of *RKDs* and *NLPs* in elephant grass under stress, RT-PCR was adopted for detecting the gene expression of *RKDs* and *NLPs* in elephant grass under salt (NaCl 100 mM), drought (PEG 20%) and high temperature(40 °C/38 °C) stress, respectively. *CpNLP5.2* in elephant grass can rapidly respond to a variety of abiotic stress signals, and its expression significantly increased after 2 h of high temperature, drought, and salt stress, indicating that *CpNLP5.2* exerts a core effect on the adaptation of elephant grasses to stress. Meanwhile, four randomly selected *RKD*s were differentially expressed under high-temperature stress, explaining that they have special effects under heat stress(Fig. 5). In addition, we found that the expressions of *CpAPX*, *CpSOD,* and *CpPOD* in elephant grass were also significantly increased under the three kinds of stresses (Fig. S3). Among them, *CpRKD*1.1, *CpRKD*4.3, and *CpRKD*6.6 were correlated with *CpPOD* expression, which means that *RKD* might be related to peroxide synthesis (Fig. S4).

### Functional of *NLP* and *RKD* of *RWP* gene family

Gene expression is closely related to function. In order to further reveal the influence of the RWP gene family expansion on grass thermal adaptability, the DAP-seq target gene information of At*NLP*7 and At*RKD*2 was provided by Plant Cistrome Database for subsequent *RWPs* functional analysis.

**Fig. 6:**
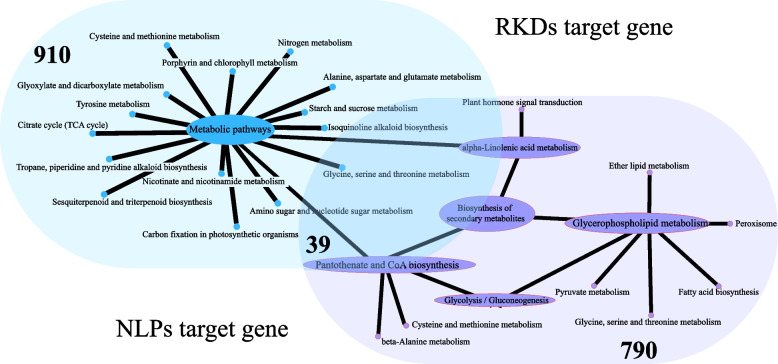
Functional annotation analysis of RKDs and NLPs downstream genes. Blue areas represent 790 target genes bound by AtRKD2 (FRIP > 0.05); The purple area shows the 910 target genes bound by AtNLP7(FRIP > 0.05); The intersection of blue and purple represents the 39 target genes shared by AtNLP7 and AtRKD2 (FRIP > 0.05)

Based on the functional annotations of At*NLP*7 target genes, not only genes taking part in the control of N metabolisms, including ferredoxin-nitrite reductase (*nirA*), NR reductase (*NR*) and NRT /nitrite (*NRT*) but also significantly enriched carbon metabolic pathways (Value = 0.002571), associated genes such as phosphoenolpyruvate carboxylase (*PPC*), malate dehydrogenase (*MDH1*) and triosephosphate isomerase (*TPI*) suggested that *NLPs* could regulate the balance between nitrogen uptake and C fixation in plants(Fig. 6, Table S5). Although some studies have shown that At*NLP*7 mutants may be involved in physiological and metabolic processes related to drought stress [20], functional annotation analysis of *AtNLP7* target genes showed that *NLPs* were more important for stable plant growth. Regulating nitrogen-nitrogen related balance-related genes nitrogen-mediated tiller growth response 5 (*NGR5*) and growth-regulating factor 4 (*GRF4*) successfully improved nitrogen application efficiency and yield in rice [21]. Therefore, it is of great significance to work on the biological function and regulatory mechanism of *NLPs* for crop improvement.

Compared with *NLPs*, the *RKDs* have been rarely studied in plants, and only a few studies have shown that *RKDs* may be related to gamete development in the plants [22]. In the functional annotation enrichment analysis results of *AtRKD2* target genes, no genes related to nitrogen metabolism were found, but the enrichment of more genes was realized in the biosynthesis of secondary metabolites, including phenylpropanoid, pantothenate and CoA biosynthesis, etc. (Fig. 6, Table S6). These secondary metabolites can assist plants to cope with abiotic stressful condition and are also often used as physiological indicators of plant response to stresses, such as peroxidase and sinapate 1-glucosyltransferase (*BRT1*). In addition, the expression of a related peroxidase gene in elephant grass under heat, drought, and salt stress was found highly upregulated in the present study, suggesting that *RKDs* played a positive role in elephant grass resisting abiotic stresses. Therefore, *RKDs* may play an important role to enhance the resistance of elephant grass against abiotic stresses besides participating in the formation and development of gametes.

## Discussion

High temperature is a typical climate characteristic in tropical and subtropical regions, especially in Africa, which enables native plants to constantly evolve in the process of adapting to the variable environments and also provides more genetic resources for the study of plant heat tolerance [3]. Whole-genome duplication (WGD) is widely present in plant genomes and is an important event that drives plant evolution [23]. WGD, also known as polyploidization, rapidly reorganizes plant genomes by losing many genes, increasing structural variations, and playing a crucial role in plant evolution and diversity [24]. WGD not only promotes the formation of elephant grass species but also provides rich genetic resources to help elephant grass adapt to a heated environment. 73% of *RWPs* production was associated with WGD, which means that WGD is the source driving force of *RWP* gene family expansion in elephant grass.

The functional adaptive evolution of the NBS_LRR gene family in plants enables them to adapt to complex natural environments [25]. The expansion of the RWP gene family in elephant grass is closely related to Africa's harsh ecological environment. The expression of *CpNLP5.2* increased significantly after 2 h of heat, drought, and salt stress, and the four randomly selected *RKD*s also increased expression under heat stress, suggesting that the *RWP* gene family exerts a core effect on the adaptation of elephant grass to heat stress. Recent studies on the function of the RWP gene of its related species Pearl millet showed that the increase of the RWP gene family was closely related to the adaptation of elephant grass to the natural environment of high temperature and drought in Africa. MeJA and ABA are two major plant hormones in response to abiotic stress and exert an important role in plant answer to abiotic stress. In this study, the promoter regions of NLP and RKD are rich in the response elements of these two hormones, In vitro addition of ABA hormone showed that ABA could rapidly induce RWP gene expression. However, the expressions of NLP and RKD were significantly different under the three different stress treatments. For example, NLP was more active to salt stress signals, while RKD was more active to heat stress. This difference in the expression of RWP gene family may result from the functional differentiation of NLP and RKD.

NLP is a core transcription factor that mainly regulates the nitrate content in plant cytoplasm [26]. The root-to-shoot nitrate allocation response (SINAR) is a key mechanism for plants to adapt to stressful environments by increasing the distribution of nitrate in roots under stress conditions [27]. When high levels of nitrate are detected, NLP is transported to the nucleus and activates the expression of genes taking part in nitrate transport and metabolism, including NRT1.1, NRT2.1, and NIA1 [28]. NRT1.5 [29] and NRT1.8 [30] can enhance the tolerance of Arabidopsis to various abiotic stresses and affect the accumulation of malondialdehyde and proline. AtNLP7 can bind to key nitrate pathway genes and regulate nitrate assimilation and metabolism by activating or inhibiting downstream transcriptional regulatory factor TCP20 [31]. AtNLP6 promotes root meristem growth under nutrient stress conditions by controlling the expression of nitrate-responsive genes [32]. Additionally, AtNLP7 can also regulate nitrate assimilation and metabolism through a similar mechanism, helping plants adapt to stressful environments [31]. Moreover, the discovery of the nitrate-CPK-NLP signaling pathway highlights the importance of NLP phosphorylation in plant vegetative growth [33].

*RKD*, another subfamily of the *RWP* gene family, was observed in this research to be mainly involved in the synthesis of Phenylpropanin secondary metabolites. These secondary metabolites are mainly polyphenolic compounds, which can remove harmful free radicals in plants and improve plant tolerance under heat, drought, and salt stress [34]. Additionally, studies have demonstrated that RKD is closely associated with gamete formation and development [35]. AtRKD1 and AtRKD2 transcripts are only detectable during the later phases of female gametophyte growth, while AtRKD3, AtRKD4, and AtRKD5 transcripts are detectable during both early and late phases, according to Tedeschi et al. [36]. AtRKD1 and AtRKD2 are highly expressed in the egg apparatus and egg cells. However, overexpressing AtRKD1 and AtRKD2 can result in cell proliferation, differentiation and regeneration defects, and a change in gene expression towards an egg cell-like transcriptome [16]. During their reproductive period, plants are particularly vulnerable to high temperatures. High temperatures can inhibit spikelet development, reduce seed set, impair rice grain filling, decrease grain weight, and affect grain quality. Therefore, RKDs may exert a key effect on plant response to heat stress, particularly during the reproductive stage.

The expansion of *RWP* gene family improves the heat tolerance of elephant grass in the two fields below: the expansion of *NLP* subfamily improves the transport and absorption of nitrate nitrogen and reduces the influence of heat stress on its development; *RKD* expansion promotes the synthesis of phenylpropanoid secondary metabolites under heat stress and reduces stress damage. Although the first RWP protein was identified in chlamydomonas as early as 1997, the functional mechanism of the *RWP* gene family in abiotic stress has been rarely studied [37]. By analyzing the evolution and function of the significantly expanded RWP gene family in elephant grass under heat stress, it was further confirmed that the conserved gene family had rich genetic diversity and functional specificity in non-model plants. Hence, analyzing the specific heat tolerance mechanism of non-model plants is crucial to understand the evolution of plant heat adaptation and reducing the impact of global warming on crop safety.

## Conclusions

The hot climate in Africa has driven the thermal adaptation evolution in elephant grass, while whole genome duplication (WGD) has facilitated the expansion of the RWP-RK gene family. Under high temperature stress, the RKD subgroup demonstrates a more rapid response compared to the NLP subgroup. This could be attributed to the significant presence of MeJA and ABA responsive elements in the promoter region of the RKD subgroup, which contribute to their active response to high temperature stress. These research findings provide a scientific basis for analyzing the heat adaptation mechanism in elephant grass and improving the heat tolerance of other crops. Further research into the heat adaptation characteristics of elephant grass will help us better address the challenges posed by climate change and provide valuable guidance for crop breeding and improvement.

## Materials and methods

### Plant materials and treatments

The elephant grass (*Pennisetum purpureum* Schum. ‘LSPR’) accessions were collected from Chongzhou, Sichuan, China. The accessions were grown in a nutrient solution under glasshouse conditions with a 14-h light and 10-h dark cycle at 28 °C and 25 °C, respectively. At the three-leaf stage (4 weeks of age), potted seedlings with similar phenotypes were selected for abiotic stress experiments. For heat treatment, elephant grass leaves were collected at 0, 6, 12, and 48 h after exposure to a temperature of 40 °C with a 14-h light and 10-h dark cycle. Drought and NaCl treatments were applied using 20% polyethylene glycol (PEG) and 100 mM NaCl solution instead of nutrient solution, respectively. Leaves were collected at 0, 2, 4, and 6 h after treatment. Each treatment was collected in triplicate per time point.

### RNA separation and quantitative real-time polymerase chain reaction analysis

The Plant Total RNA Isolation Kit (FOREGENE, Chengdu, China) was adopted to collect elephant grass leaves and extract total RNA, followed by reverse transcription with the HiScript III 1st Strand cDNA Synthesis Kit. Gene expression was normalized with elongation factor-1-alpha (EF1α) as the reference gene. qRT-PCR was made with the CFX96 qRT-PCR System and ChamQ Universal SYBR qPCR Master Mix, with the reaction program following the standard protocol of the kit. Through comparing the relative expression level of target genes, the calculation of control treatments was made with the 2 − ΔΔCt approach. Primer Premier 5.0 software was adopted to design quantitative real-time polymerase chain reaction (qRT-PCR) primers, and Table S4 lists the qRT-PCR primer sequences.

## Identification of *RWP* genes

For identifying members of the RWP gene family in each species, the potential Markov model of RWP was offered by PFAM (http://pfam.janelia.org/, [38]). Candidate genes were then verified using NCBI CD-search (https://www.ncbi.nlm.nih.gov/Structure/cdd/wrpsb.cgi, [39]) to confirm their conservative structure domain (E-value < 0.01).

## Multiple-sequence alignment and phylogenetic analysis

For comparing full-length protein sequences across each species, a sequence alignment was created using the MUSCLE tool (http://www.ebi.ac.uk/Tools/msa/muscle/) and saved in ClustalW format. Subsequently, a phylogenetic analysis was performed with 1000 bootstrap replications with the MEGA 6.0 program [40]. The Poisson model method and the same alignment file were adopted to construct an unrooted Neighbor-joining and Minimum-Evolution tree.

### Comparison of chromosomal distributions, exon/intron structures, and protein domains in elephant grass

For the analysis of the genomic distribution of RWP genes in elephant grass, we utilized MapInspect software (https://mapinspect1.software.informer.com/, [41]). Additionally, we employed the Gene Structure Display Server 2.0 program (http://gsds.cbi.pku.edu.cn/) to examine intron/exon structures. To predict RWP-RK domains, we used the MEME (Multiple Expectation Maximization for Motif Elicitation) online tool (http://meme-suite.org/tools/meme) with the parameters motif width 6–200 residues and a maximum number of motifs = 20 [42]. Subsequently, we downloaded the conserved motif logos from MEME, and we generated motif images with TBtools_master (version 1.098769; https://github.com/CJ-Chen/TBtools).

### Genomic collinearity analysis

Tandem duplications were detected in elephant grass and sorghum genomes using BLAST and MCScanX. Multilocus genes were classified as tandem duplicates if they were in nearby areas or separated by uniform intergenic areas, with coverage and similarity levels above 90% and 95%, respectively.

### Analyze of the promoter cis-regulatory elements

To analyze potential cis-acting regulatory factors in the putative promoter areas of elephant grass RWPs, the 2-kb sequences upstream of each gene were acquired through BLAST searches of the elephant grass genome with whole gene IDs. The PlantCARE database (http://bioinformatics.psb. ugent.be/webtools/plantcare/html/, [43]) was adopted to analyze the obtained sequences for cis-elements identification. A custom script in R program version 4.1.0 (https://www.r-project.org/) was adopted to visualize the identified cis-elements.

### Biophysical properties of *RWP* genes in elephant grass

To determine the physical and chemical properties of the RWP proteins in the elephant grass genome, their amino acid number, molecular weight, and theoretical pI were calculated with the ExPASy-ProtParam tool [44]. Furthermore, the subcellular localization of these proteins was forecast with ProComp 9.0 [45].

### Gene expression patterns analyzed using published transcriptome data

Raw transcriptome data were processed with reference to the analysis by Di Bella et al., [46] and the log10 transformed Transcripts Per Kilobase Million fragments (TPM) mapped values of the 36 RWP genes were calculated. Heatmaps were generated using the local Multiple Arrays Viewer program (https://sourceforge.net/projects/mev-tm4/files/mev-tm4/).

### Statistical analysis

The statistical analysis of data was made with one-way analysis of variance (ANOVA) and significance was determined with Dunnett’s test with a p-value threshold of 0.05. Differential expression was evaluated with a two-fold cut-off value to consider the biological significance of the observed changes [47]. To investigate the relationship between *CpPOD* and *CpRKD* (*CpRKD1.1*, *CpRKD4.3*, and *CpRKD6.6*), the calculation of Pearson correlation coefficients was made. All statistical analyses were made with R version 3.6.1.

### KEGG annotation analysis of *AtNLP7* and *AtRKD2* target genes

Firstly, potential regulators of target genes were identified from the Plant Cistrome Database [48] based on a FRIP score of > 0.05, with AtNLP7 and AtRKD2 identified as potential transcription factors. Subsequently, functional enrichment analysis was made on the identified target genes with KOBAS (KEGG Orthology Based Annotation System, http://kobas.cbi.pku.edu.cn/, [49]). The resulting *p*-values were corrected for FDR, with a significance threshold of *p* ≤ 0.05, to determine significantly enriched pathways related to peak-related genes.

### Supplementary Information

**Additional file 1:** **Figure S1.** Phylogenetic analysis of RKD and NLP subfamilies of Elephant grass RWP gene family.(the distance scale is 0.9). **Figure S2.** Mechanism analysis of RWP gene family expansion in elephant grass.(A: Collinearity analysis of RWP gene in elephant grass and sorghum genome; B: Chromosome mapping of RWP gene in elephant grass). **Figure S3.** Expression trend of CpAPX、CpSOD and CpPOD in elephant grass under abiotic stress(*:*p*<0.05; **: *p*<0.01). A:salt(NaCl 100mM; B:drought(PEG 20%); C: heat (40°C/38°C, 12h light / 12h dark). (*:*p*<0.05 ; ** *p*<0.01). **Figure S4.** CpRKD1.1, CpRKD4.3, and CpRKD6.6 were correlated with CpPOD expression.

**Additional file 2:** **Table S1.** Ka/Ks analysis of homologous genes between Elephant grass and sorghum.

**Additional file 3:** **Table S2.** Expression pattern analysis of RWPs in 5 tissues of Elephant grass.

**Additional file 4:** **Table S3.** Analysis of RWPs promoter hormone-responsive elements in elephant grass.

**Additional file 5:** **Table S4.** The primer of RWPs in elephant grass for qRT-PCR.

**Additional file 6:** **Table S5.** KEGG functional annotation enrichment analysis of 910 target genes bound by AtNLP7.

**Additional file 7:** **Table S6.** KEGG functional annotation enrichment analysis of 790 target genes bound by AtRKD2.

## Data Availability

The following sources provided the genome and protein sequence for rice, elephant grass, pearl millet, and sorghum: https://ftp.ebi.ac.uk/ensemblgenomes/pub/release-56/plants/fasta/oryza_sativa/dna ([50] rice), http://milletdb.novogene.com/home/ ([51], elephant grass, CIAT6263), https://www.ncbi.nlm.nih.gov/bioproject/?term=PRJNA749489 ([52], pearl millet, PRJNA749489), and https://www.ncbi.nlm.nih.gov/bioproject/?term=PRJEB24962 ([53], sorghum, PRJEB24962). To investigate the expression patterns of RWPs in different vegetative tissues of elephant grass, we utilized high-throughput transcriptome sequencing data from PRJNA649119 (https://www.ncbi.nlm.nih.gov/bioproject/649119, [54]). The potential regulators of AtNLP7 and AtRKD2 target genes raw data download from http://neomorph.salk.edu/dap_web/pages/browse_table_aj.php. The data used to support the findings of this study are included within the main text and supplementary files of this article.
