# Supplementary material for: The evolution and expansion of RWP-RK gene family improve the heat adaptability of elephant grass (Pennisetum purpureum Schum.)
Source: BMC Genomics. 2023 Aug 31;24:510. doi: 10.1186/s12864-023-09550-8 (PMC10472707; doi:10.1186/s12864-023-09550-8)
Supplement: Supplementary file 1 — Additional file 1: Figure S1. Phylogenetic analysis of RKD and NLP subfamilies of Elephant grass RWP gene family.(the distance scale is 0.9). Figure S2. Mechanism analysis of RWP gene family expansion in elephant grass.(A: Collinearity analysis of RWP gene in elephant grass and sorghum genome; B: Chromosome mapping of RWP gene in elephant grass). Figure S3. Expression trend of CpAPX、CpSOD and CpPOD in elephant grass under abiotic stress(*:p<0.05; **: p<0.01). A:salt(NaCl 100mM; B:drought(PEG 20%); C: heat (40°C/38°C, 12h light / 12h dark). (*:p<0.05 ; ** p<0.01). Figure S4. CpRKD1.1, CpRKD4.3, and CpRKD6.6 were correlated with CpPOD expression. [file 12864_2023_9550_MOESM1_ESM.docx]

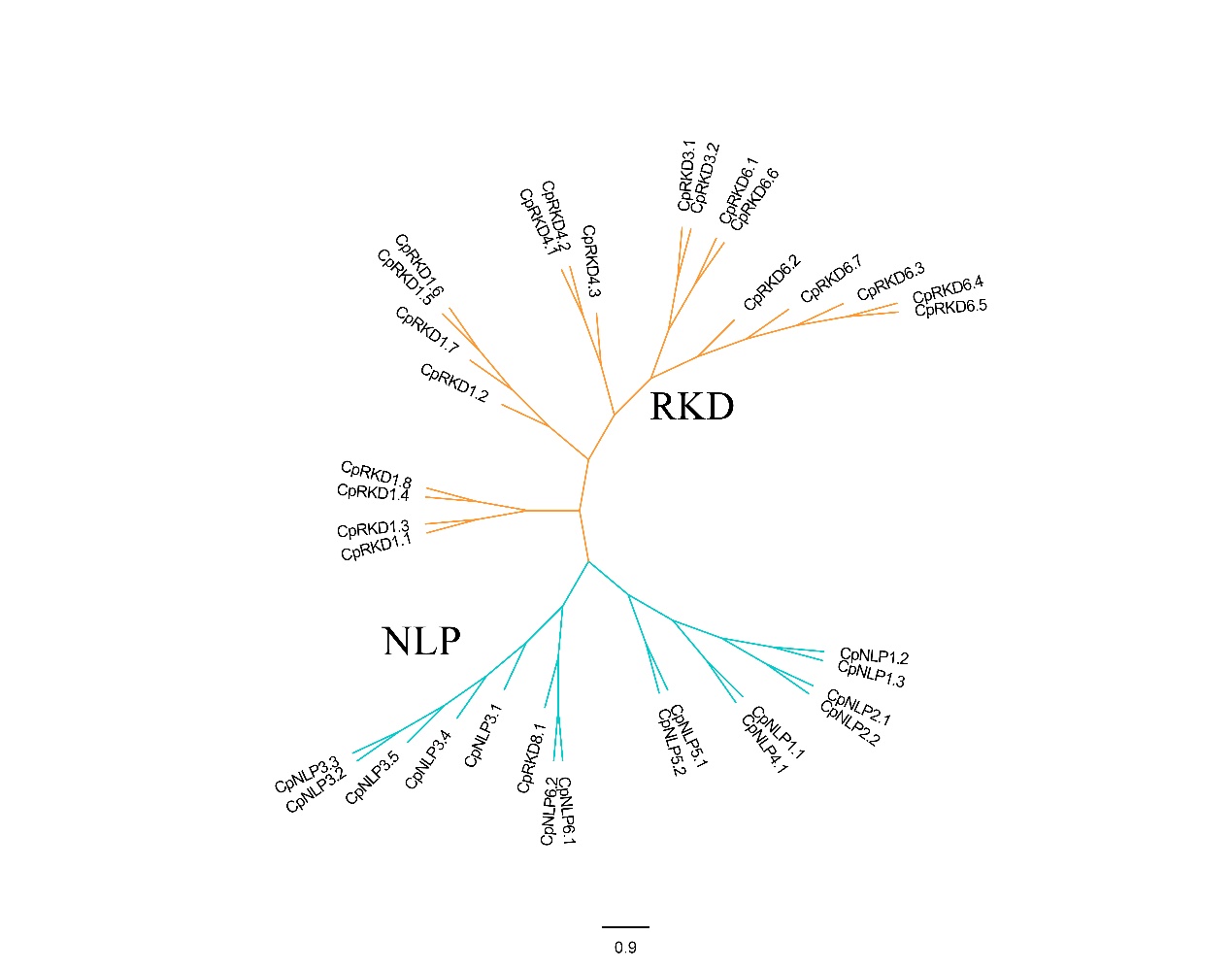


Figure S1. Phylogenetic analysis of RKD and NLP subfamilies of Elephant grass RWP gene family.(the distance scale is 0.9)


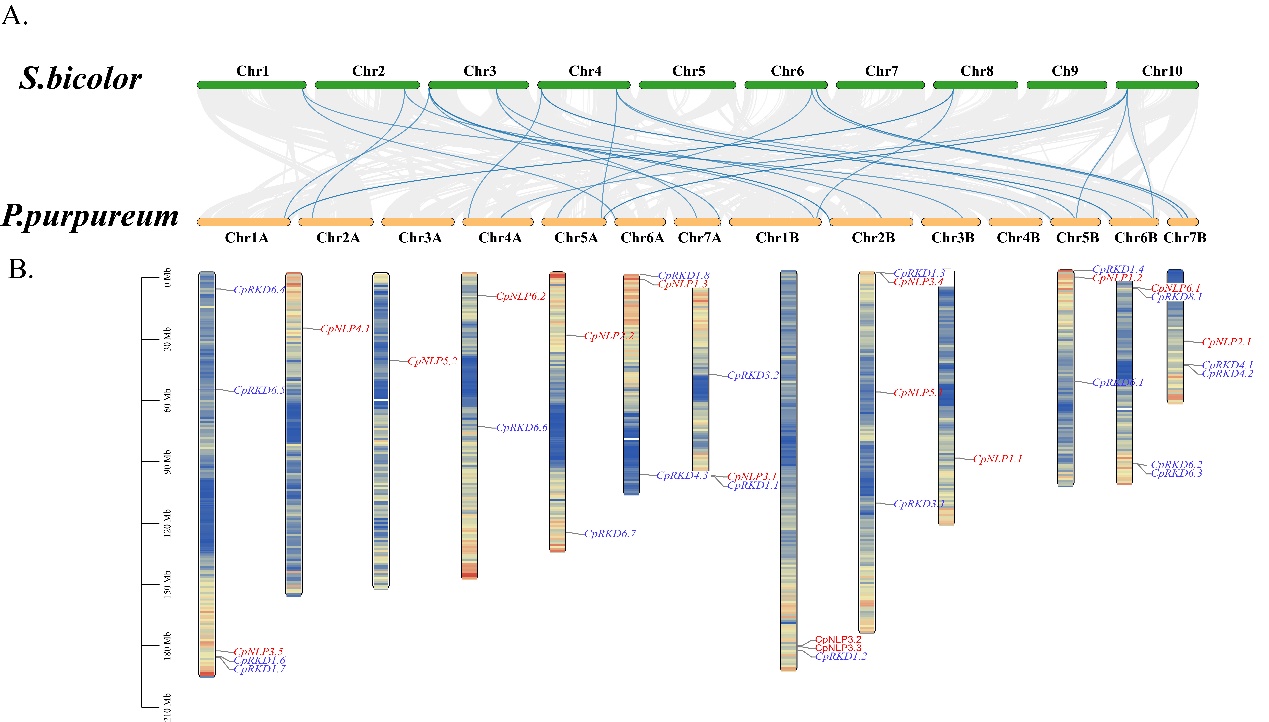


Figure S2. Mechanism analysis of RWP gene family expansion in elephant grass.(A: Collinearity analysis of RWP gene in elephant grass and sorghum genome; B: Chromosome mapping of RWP gene in elephant grass)


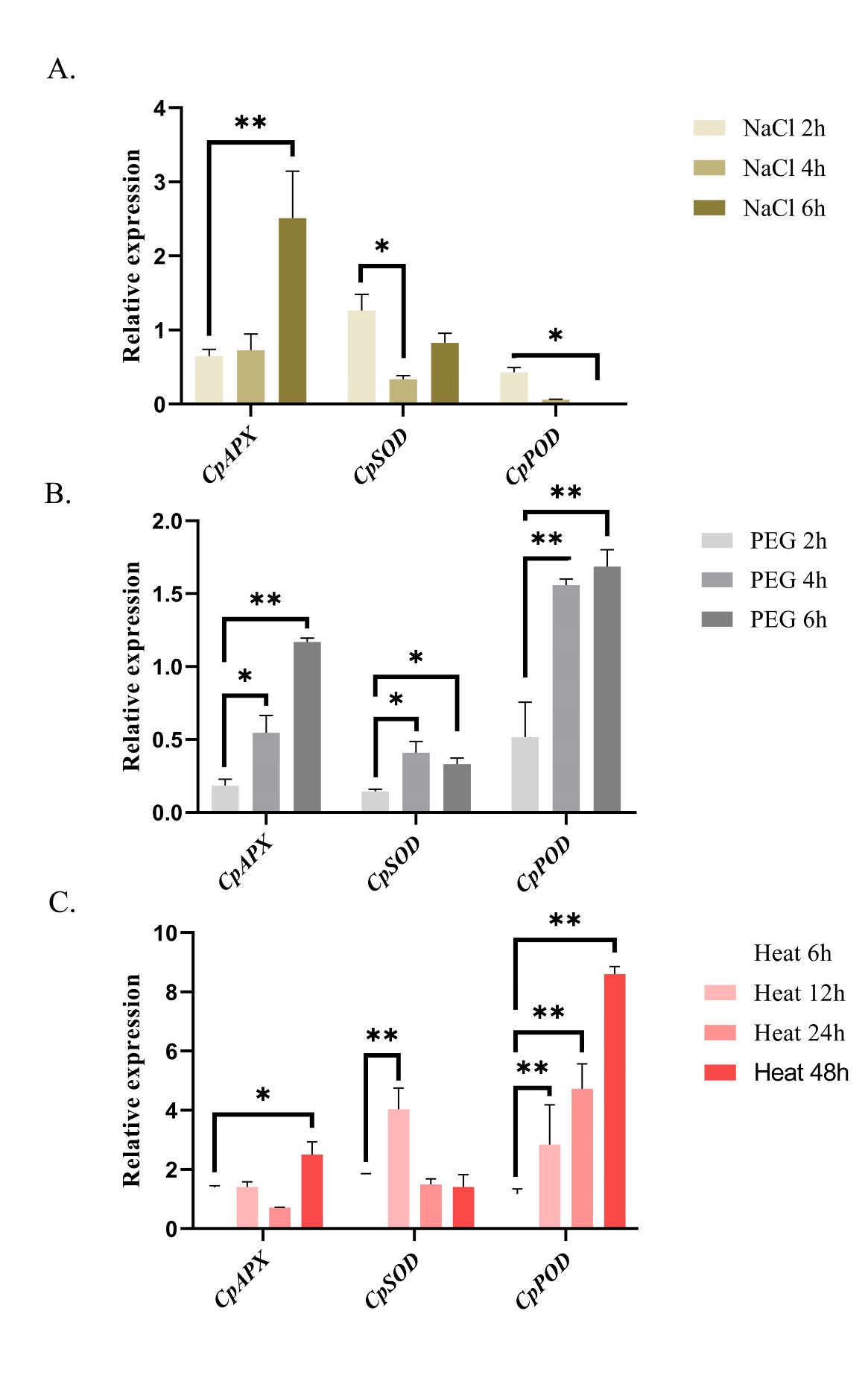


Figure S3. Expression trend of *CpAPX*、*CpSOD* and *CpPOD* in elephant grass under abiotic stress(*:p<0.05; **: p<0.01). A:salt(NaCl 100mM; B:drought(PEG 20%); C: heat (40°C/38°C, 12h light / 12h dark). (*:p<0.05 ; ** p<0.01)


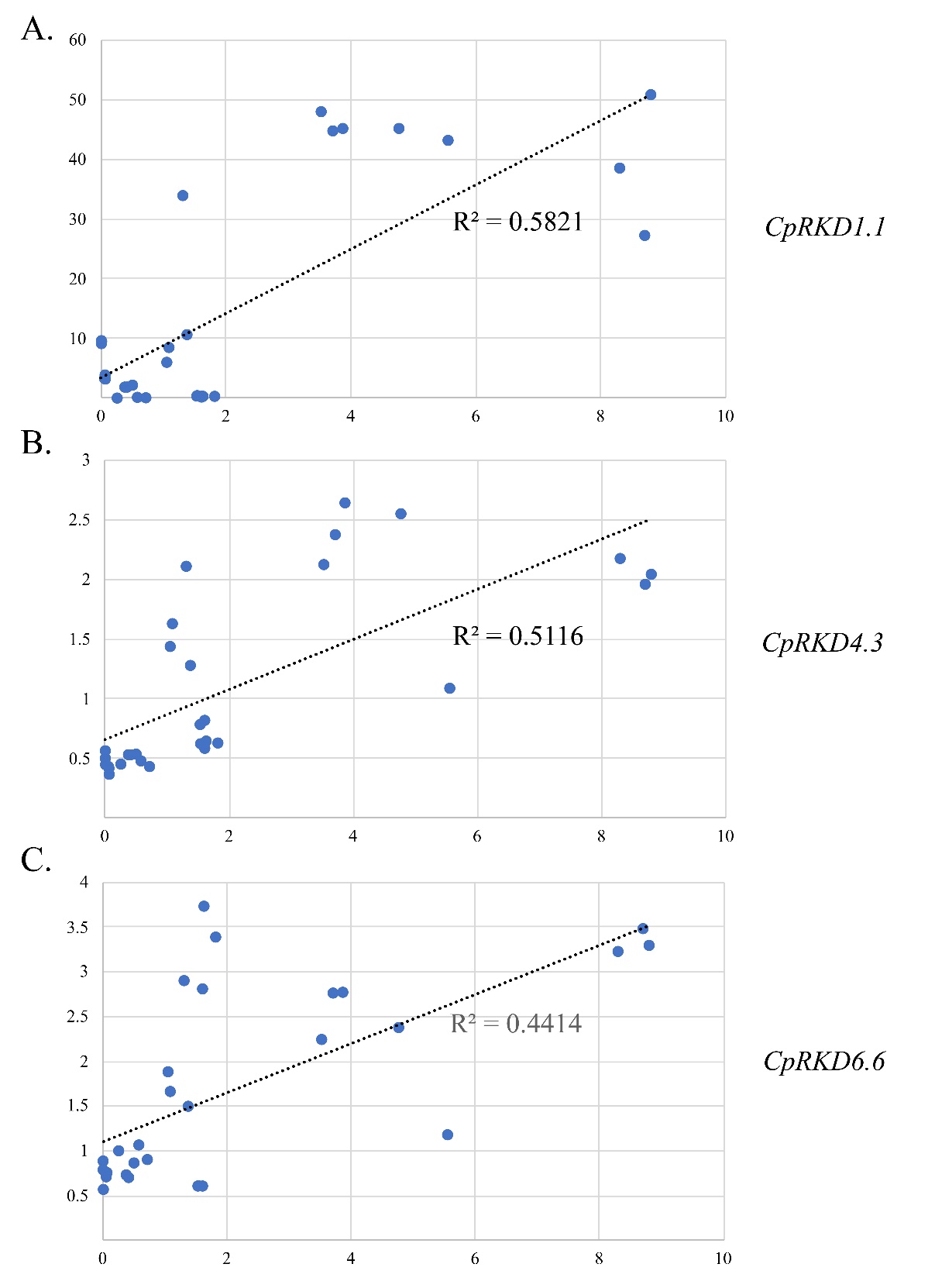


Figure S4. CpRKD1.1, CpRKD4.3, and CpRKD6.6 were correlated with CpPOD expression
